# Enhancing the reactivity of 1,2-diphospholes in cycloaddition reactions

**DOI:** 10.3762/bjoc.11.17

**Published:** 2015-01-27

**Authors:** Almaz Zagidullin, Vasili Miluykov, Elena Oshchepkova, Artem Tufatullin, Olga Kataeva, Oleg Sinyashin

**Affiliations:** 1 A. E. Arbuzov Institute of Organic and Physical Chemistry, Kazan Scientific Centre, Russian Academy of Sciences, Arbuzov Str. 8, 420088 Kazan, Russia

**Keywords:** cycloaddition, phospholes, phosphorus heterocycles, polycyclic phosphines, retro-Diels–Alder reaction

## Abstract

Two different approaches have been employed to enhance the reactivity of 1-alkyl-1,2-diphospholes – the introduction of electron-withdrawing groups either at the phosphorus atoms or in the para-position of the arene ring. The alkylation of sodium 1,2-diphospha-3,4,5-triphenylcyclopentadienide with alkyl halides Hal-CH_2_-R (R = CN, COOEt, OMe, CH_2_OEt) results in corresponding 1-alkyl-3,4,5-triphenyl-1,2-diphospholes (alkyl = CH_2_CN (**1a**), CH_2_COOEt (**1b**), CH_2_OMe (**1c**), and (CH_2_)_2_OEt (**1d**)), which spontaneously undergo the intermolecular [4 + 2] cycloaddition reactions at room temperature to form the mixture of the cycloadducts, **2a**–**c**, respectively. However the alkylation of sodium 1,2-diphospha-3,4,5-tri(*p-*fluorophenyl)cyclopentadienide with ethyl iodide leads to stable 1-ethyl-3,4,5-tris(*p*-fluorophenyl)-1,2-diphosphole (**1e**), which forms the [4 + 2] cycloadduct 2,3,4,4a,5,6-hexa(*p*-fluorophenyl)-1-ethyl-1,7,7a-triphospha-4,7-(ethylphosphinidene)indene (**2e**) only upon heating up to 60 °C. With further heating to 120 °C with *N*-phenylmaleimide, the cycloadducts **2a–c** and **2e** undergo the retro-Diels–Alder reaction and form only one product of the [4 + 2] cycloaddition reaction **3a–с**, **3e** with good yields up to 65%.

## Introduction

Phospholes are weakly aromatic heterocycles and demonstrate rather different properties from those of their S, N and O counterparts [1–2]. Due to low their aromaticity, phospholes are of significant interest for the preparation of highly effective catalysts, materials for light-emitting diodes and nonlinear optics [3–4]. In contrast to furans, thiophenes and pyrroles, phospholes display cycloaddition and complexation reactions and can be used as starting materials for caged phosphines, phosphinidenes, etc. [2]. At the same time, the presence of electron-withdrawing substituents (cyano-, alkoxy-, or halo-) at the phosphorus atom reduces the aromaticity of the monophosphole ring and facilitates cycloaddition reactions resulting in novel 7-phosphanorbornenes [5–6], which was verified by theoretical calculations and experimental work [7–8].

At the same time both the presence of the P=C bond in phospholes as well as the transient 2*H*-phospholes [3] increase the cycloaddition reactivity. It was previously demonstrated that 1-alkyl-1,2-diphospholes combine the properties of both 1*H*-phospholes (with thermal stability up to 190 °C) and 2*H*-phospholes (exhibiting high reactivity in the cycloaddition reaction at 25 °C) [9–11]. In the present work, attempts to increase the reactivity of the dienic system of 1,2-diphospholes using two different approaches are described: (a) by the introduction of electron-withdrawing groups (EWGs) at the phosphorus atom or (b) the introduction of EWGs to the carbon atoms of aryl substituents. This work will provide access to new polycyclic, organophosphorus compounds having significant potential as weak, bulky ligands in homogeneous catalysis [12–14].

## Results and Discussion

**Scheme 1:**
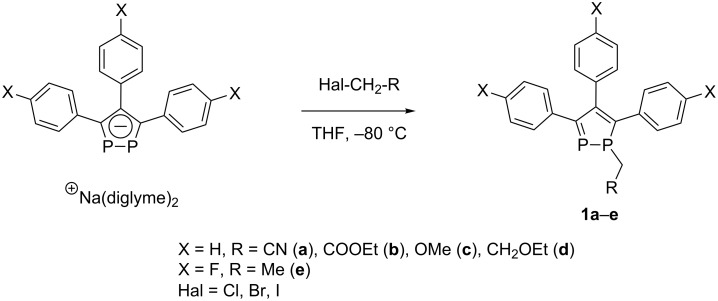
Synthesis of 1-alkyl-1,2-diphospholes **1a–e**.

The 1-alkyl-1,2-diphospholes **1a–e**, incorporating EWGs at the phosphorus atom or in aromatic fragments, are easily accessible by the alkylation of sodium 1,2-diphospha-3,4,5-triarylcyclopentadienide with alkyl halides. The reactions were carried out in THF at −80 °C with yields of 55–60% for **1a**–**e** (Scheme 1).

The structures of the obtained compounds, **1a–e**, were unambiguously confirmed by ^31^Р, ^1^Н and ^13^С NMR spectroscopy. The ^31^P NMR spectra of **1a–e** (Table 1) showed two doublets in the range of 30–60 and 210–225 ppm, corresponding to three- and two-coordinated phosphorus atoms, respectively, with a large coupling constant ^1^*J*_PP_ ≈ 365–410 Hz, which is typical for 1-alkyl-1,2-diphospholes [15].

**Table 1:** ^31^Р {^1^H} NMR spectral data of new 1-R-1,2-diphospholes **1a–e**.

	δ_P_, ppm	Δδ_P_, ppm	^1^*J*_PР_, Hz	R	X

**1a**	225.7 (P_1_=C) and 30.8 (P_2_)	194.9	363.1	CN	H
**1b**	223.3 (P_1_=C) and 40.5 (P_2_)	182.8	389.0	COOEt	H
**1c**	209.3 (P_1_=C) and 61.2 (P_2_)	148.1	404.0	OMe	H
**1d**	214.1 (P_1_=C) and 51.9 (P_2_)	162.2	407.3	CH_2_OEt	H
**1e**	218.5 (P_1_=C) and 73.4 (P_2_)	145.1	407.8	Me	F

Remarkably, the coupling constants ^1^*J*_PР_ increase with the decrease of the electron-withdrawing properties of the substitutents in the diphosphole ring (CN > COOEt > OMe > CH_2_OEt) in the series **1a–1d**.

A large phosphorus–phosphorus coupling constant, ^1^*J*_PР_, usually indicates significant σ–π delocalization of the lone pair of the tricoordinated phosphorus atom into the diphosphole ring system. Thus, the ^1^*J*_PP_ coupling constant for a non-aromatic 1,2-diphosphacyclopentene is observed at around 220 Hz [16], although both phosphorus atoms of the highly aromatic 1-(2,4,6-tri-*tert*-butylphenyl)-1*H*-1,2-diphosphole are coupled with a larger phosphorus–phosphorus constant (^1^*J*_PР_ = 528.2 Hz) [17]. The same phenomena was noted for the 1,2,4-triphosphole with the planar tricoordinated phosphorus [18]. Thus, the increase of ^1^*J*_PР_ in a sequence from **1a** to **1d** could imply the increasing delocalization of the RP-fragment within the diphosphole system that reflects the stability and the reactivity of 1,2-diphosphole.

**Scheme 2:**
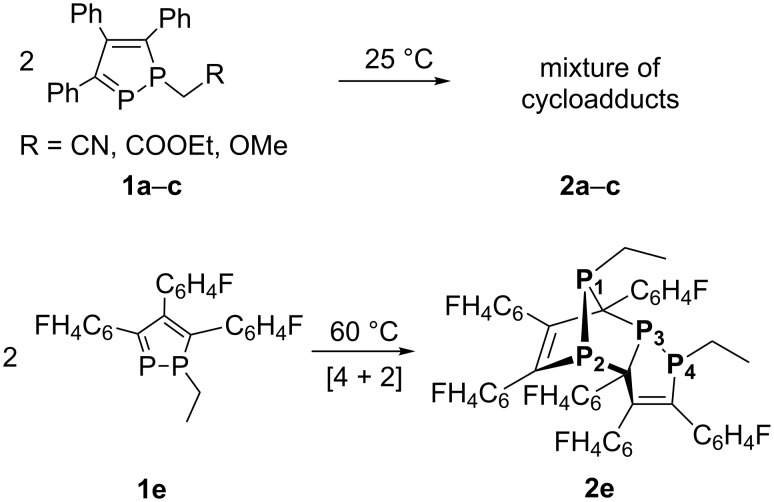
The cycloaddition reactions of 1-alkyl-1,2-diphospholes **1a**–**e**.

Indeed, the compounds **1a**,**b** are stable only at temperatures below +5 °C, while **1c** is stable at room temperature for a few hours. 1,2-Diphosphole **1d** is more stable and no cycloaddition was observed upon heating in toluene. Upon standing, the diphospholes **1a–c** undergo spontaneous [4 + 2] cycloaddition reactions leading to a mixture of cycloadducts (Scheme 2). The ^31^P NMR spectra of the reaction mixtures showed many multiplets at 80 and −40 ppm with a coupling constant ^1^*J*_PP_ ca. 200–210 Hz characteristic for the products of [4 + 2] cycloaddition reaction – 1,7-diphosphanorbornadienes [9,11]. Remarkably, 1-alkyl-1,2-diphospholes without EWGs reveal significant thermal stability and dimerization was observed only upon heating to 190 °C leading to the product of the [2 + 2] cycloaddition reaction [9]. At the same time, 3,4,5-tri(*p*-fluorophenyl)-1-ethyl-1,2-diphosphole (**1e**) is stable at room temperature and undergoes the [4 + 2] cycloaddition reaction only upon heating at 60 °C resulting in only one product, **2e** (Scheme 2).

**Figure 1:**
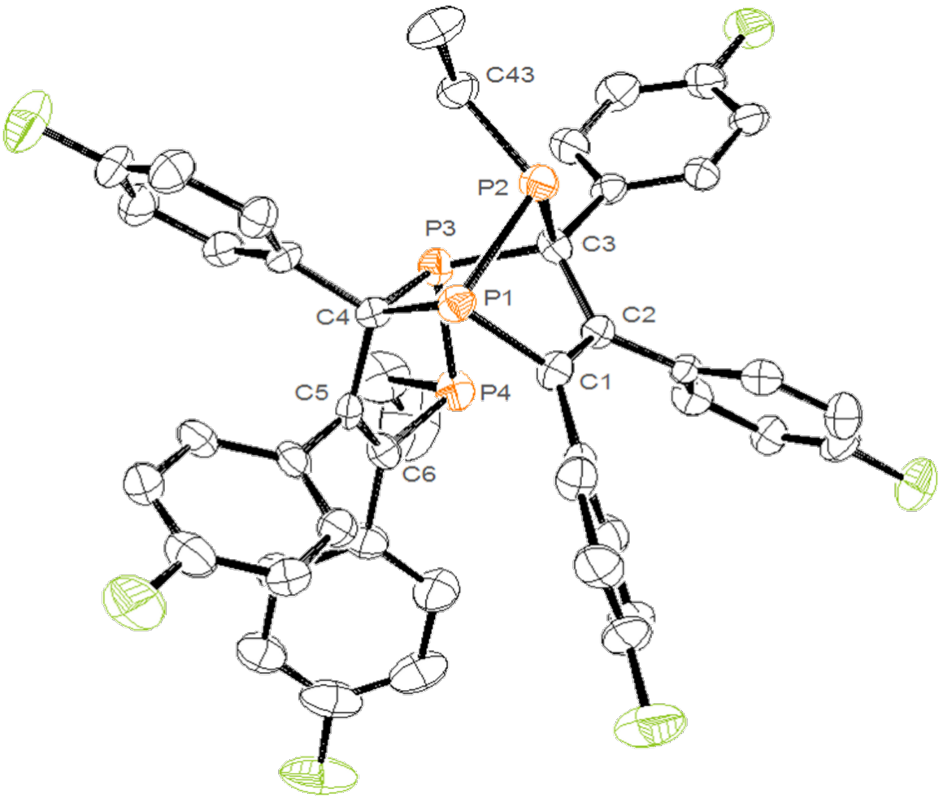
ORTEP view of 2,3,4,4a,5,6-hexa(*p*-fluorophenyl)-1-ethyl-1,7,7a-triphospha-4,7-(ethylphosphinidene)indene (**2e**). Hydrogen atoms are omitted for clarity. Selected bond lengths [Å] and angles [°]: P1–C1 1.850(5); P1–С4 1.923(6); P1–Р2 2.213(2); P2–С43 1.843(5); P2–С3 1.896(5); C1–С2 1.360(7); C2–С3 1.540(7); P3–С3 1.924(6); P3–С4 1.902(6); P3–P4 2.193(2); P4–С6 1.832(5); C5–С6 1.340(7); C5–С4 1.539(7); C1–P1–C4 97.9(2); C1–P1–P2 85.79(18); C4–P1–P2 99.14(17); C43–P2–C3 105.5(2); C43–P2–P1 109.9(2); C3–P2–P1 87.07(17); C4–P3–C3 100.1(2); C4–P3–P4 95.44(17); C3–P3–P4 101.35(17); C6–P4–C45 101.6(3);C6–P4–P3 93.40(18); C45–P4–P3 100.8(2).

The molecular structure of **2e** (Figure 1) was verified by X-ray crystallography. The crystal structure analysis of **2e** showed that only the *endo* isomer was formed with the alkyl group in *anti*-orientation with respect to the double bond of the ring.

In the case of **2a–c**, it would be assumed that similar [4 + 2] cycloadducts are formed according to the range of signals in the ^31^P NMR spectra. However, in this case, several stereoisomers are formed due to the high reactivity of the 1,2-diphospholes containing EWGs on the phosphorus atom. It should be noted that this is the first example of [4 + 2] cycloaddition between two diphosphole molecules where 1,2-diphosphole acts as a diene and a dienophile in one reaction. Therefore, these isomeric cycloadducts, **2a–с**, can be a source of reactive 1,2-diphospholes containing EWGs **1a–с** in the retro-Diels–Alder reaction [19].

**Scheme 3:**
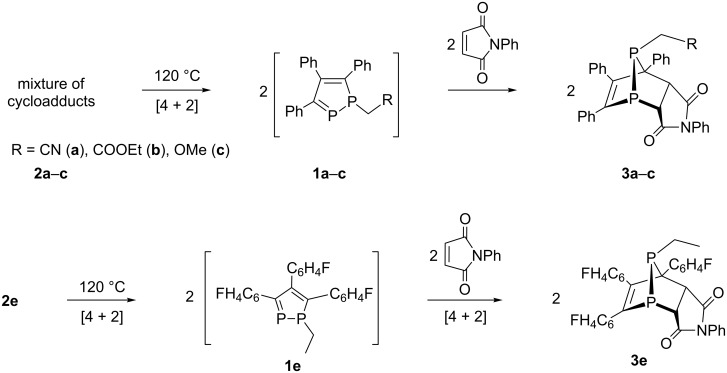
The retro-Diels–Alder reactions of the cycloadducts **2a–с**, and **2e**.

Indeed, upon further heating up to 120 °C, the mixture of the cycloadducts **2a–c** as well the cycloadduct **2e** underwent the retro-Diels–Alder reaction to form monomeric 1,2-diphospholes, **1a–c**, **1e**, which were trapped by *N*-phenylmaleimide to form the compounds **3a–c**, **3e** with yields of 55–65% (Scheme 3).

After heating for 25–30 hours at 120 °C in toluene, the ^31^Р NMR spectra of the reaction mixture displayed only two doublets at around −25 and 60 ppm with ^1^*J*_PP_ = 200 Hz. This indicates the high stereoselectivity of this reaction. The ^1^H NMR spectra of the reaction mixtures show only two doublets for protons of the *N*-phenylmaleimide fragment in the range of 4.6–4.8 ppm. Based on our previous results [20], we can conclude that only an *anti-endo-*isomer was formed in each case.

## Conclusion

In summary, we have demonstrated the prospect of increasing the reactivity of 1,2-diphospholes using two different approaches: (a) introduction of EWGs at the phosphorus atom and (b) introduction of EWGs at the carbon atoms of aryl substituents. New 1-alkyl-1,2-diphospholes, **1a–c**, **1e**, containing EWGs demonstrated high reactivity and underwent intermolecular [4 + 2] cycloaddition reactions at 25–60 °C leading to a single product, **2e**, or a mixture of [4 + 2] cycloadducts **2a–c**. Additionally, upon further heating up to 120 °C with *N*-phenylmaleimide, the mixture of isomeric cycloadducts **2a–c**, **2e** underwent the retro-Diels–Alder reaction, yielding only one product of [4 + 2] cycloaddition **3a–c**, and **3e**. The same chemical behavior was observed for 1-alkyl-1,2-diphosphole-1-oxides, which underwent [4 + 2] cycloaddition at 25 °C and the retro-Diels–Alder reaction at 100 °C [21]. Compared with 1-alkyl-1,2-diphospholes, the new 1-R-1,2-diphospholes **1a–c**, **1e** containing EWGs were less stable. Given that they are more reactive in cycloaddition reactions, this work presents the opportunity for new polycyclic phosphines.

## Supporting Information

File 1 Experimental procedures and characterization data.
