# Supplementary material for: Enhancing the reactivity of 1,2-diphospholes in cycloaddition reactions
Source: Beilstein J Org Chem. 2015 Jan 27;11:169–73. doi: 10.3762/bjoc.11.17 (PMC4362055; doi:10.3762/bjoc.11.17)
Supplement: File 1 — Experimental procedures and characterization data. [file Beilstein_J_Org_Chem-11-169-s001.pdf]

Supporting Information

for

Enhancing the reactivity of 1,2-diphospholes in cycloaddition reactions

Almaz Zagidullin*§, Vasili Miluykov, Elena Oshchepkova, Artem Tufatullin, Olga Kataeva and Oleg Sinyashin

Address: A. E. Arbuzov Institute of Organic and Physical Chemistry, Kazan Scientific Centre, Russian Academy of Sciences, Arbuzov Str. 8, 420088 Kazan, Russia

Email: Almaz Zagidullin - zagidullin@iopc.ru;

§Phone +7-843-273-93-44

*Corresponding author

Experimental procedures and characterization data

Experimental

General

All reactions and manipulations were carried out under dry pure N_2 using standard Schlenk apparatus. All solvents were distilled from sodium/benzophenone and stored under nitrogen before use. The NMR spectra were recorded on a Bruker MSL-400 (^{1}H 400 MHz, ^{31}P 161.7 MHz, ^{13}C 100.6 MHz). $SiMe_4$ was used as internal reference for ^{1}H and ^{13}C NMR chemical shifts, and 85% H_3PO_4 as external reference for ^{31}P. Infrared (IR) spectra were recorded on a Bruker Vector-22 spectrometer. The elemental analyses were carried out at the microanalysis laboratory of the Arbuzov Institute of Organic and Physical Chemistry, Russian Academy of Sciences.

X-ray Structure Determination

Data set for single crystal **2e** was collected on a Bruker AXS Smart APEX-II CCD diffractometer with graphite-monochromated Mo Kα radiation (λ= 0.71073 Å). Programs used: data collection APEX2 [1], data reduction SAINT [2], absorption correction SADABS version 2.10 [3], structure solution SHELXS97 [4], structure refinement by full-matrix least-squares against F2 using SHELXL-97 [4]. Hydrogen atoms were placed into calculated positions and refined as riding atoms in unequivocal cases. The figures were generated using ORTEP-3 [5] and Mercury CSD 2.0 [6] programs. One should note that because of the poor quality of crystals (with the ratio of reflections greater than $I > 2\sigma(I)$ only 25%) large value of Rint is observed, however the structure of this compound was determined unambiguously. It was impossible to find a reasonable model for solvate diethyl ether, thus the 'Squeeze' option of "Platon" [7] was applied to obtain a solvent-free reflection file which was further used for the refinement. **Crystal data for 2e**: formula $C_{46}H_{34}F_6P_4$+$C_4H_{10}O$, crystal size 0.18 × 0.18 × 0.11 mm^3, M = 898.73, monoclinic, space group $C2/c$, a = 22.97(1) Å, b = 10.083(5) Å, c = 37.12(5) Å,

β = 101.98(1)°, V = 8412(8) Å^3 , Z =8, $\rho_{calcd.}$ = 1.419 g cm^{-3}, μ = 0.246 mm^{-1}, θ_{max} = 28°, reflections collected: 61021; independent: 10390 (R_{int} = 0.2418) and 3841 observed reflections [I>2σ(I)], 507 refined parameters, R_1 = 0.0845, wR_2 = 0.1734 [I>2σ(I)]; max/min residual electron density: 0.39/- 0.45 e/Å^3, GoF = 0.85.

CCDC 1029752 contain the supplementary crystallographic data for this paper. These data can be obtained free of charge via www.ccdc.cam.ac.uk/conts/retrieving.html (or from the Cambridge Crystallographic Data Centre, 12, Union Road, Cambridge CB2 1EZ, UK; fax: +44 1223 336033; or deposit@ccdc.cam.ac.uk).

Materials

Sodium 3,4,5-triaryl-1,2-diphosphacyclopentadienides were obtained according to a literature procedure [8,9]. Chloroacetonitrile, ethyl-2-chloroacetate, chloro(methoxy)methane, 1-bromo-2-ethoxyethane, ethyl iodide were freshly distilled before their use. *N*-phenylmaleimide was purchased from Aldrich and used without additional purification.

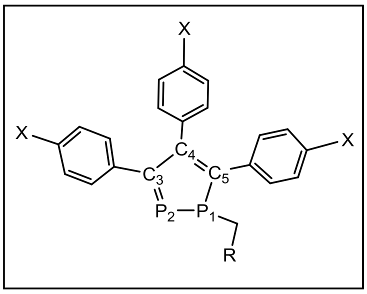

2-(3,4,5-Triphenyl-1,2-diphosphacyclopenta-2,4-dienyl)acetonitrile (1a). Freshly distilled chloroacetonitrile $ClCH_2CN$ (0.50 g, 6.55 mmol, 25% excess) was added to a solution of sodium (diglyme)$_2$ 3,4,5-triphenyl-1,2-diphosphacyclopentadienide (3.25 g, 5.24 mmol) in THF (30 mL) at –80 °C and then stirred for 2 h without cooling bath. Then the solvent was evaporated under reduced pressure and the residue was extracted with petroleum ether (2×40 mL). The petroleum ether extract was evaporated in vacuo to leave 1.06 g (55%) of 1-cyanomethylene-3,4,5-triphenyl-1,2-diphosphacyclopenta-2,4-diene (**1a**) as light-yellow oil. ^{1}H NMR (5°C, $CDCl_3$): 2.12-2.19 (m, 2H, CH_2), 6.96 (t, 3H, $^3J_{HH}$ = 6.4), 6.93-7.00 (m, 3H), 7.03 (d, 2H, $^3J_{HH}$ = 5.9), 7.07 (d, 4H, $^3J_{HH}$ = 7.3), 7.23-7.29 (m, 2H), 7.31

(t, 1H, $^3J_{HH}$ = 8.3). ^{31}P NMR (5°C, $CDCl_3$): 30.8 (d, $^1J_{PP}$ = 363.1), 225.7 (d, $^1J_{PP}$ = 363.1). ^{13}C NMR (5°C, $CDCl_3$): 17.9 (dd, $^1J_{CP}$ = 16.1, $^2J_{CP}$ = 5.8, CH_2), 115.4 (d, $^2J_{CP}$ = 6.4, CN), 126.4 (s, *p*-C_{Ph}), 126.5 (s, *p*-C_{Ph}), 126.8 (s, *p*-C_{Ph}), 127.5 (s, *m*-C_{Ph}), 128.0 (s, *m*-C_{Ph}), 128.3 (s, *m*-C_{Ph}), 128.6 (dd, $^3J_{CP}$ = 12.4, $^4J_{CP}$ = 1.5, *o*-C_{Ph}), 129.2 (d, $^3J_{CP}$ = 9.3, *o*-C_{Ph}), 131.3 (t, $^3J_{CP}$ = 9.1, *o*-C_{Ph}), 137.3 (d, $^4J_{CP}$ = 5.3, *ipso*-C_{Ph}), 138.3 (dd, $^2J_{CP}$ = 10.3, $^3J_{CP}$ = 4.1, *ipso*-C_{Ph}), 142.9 (d, $^2J_{CP}$ = 19.0, *ipso*-C_{Ph}), 150.3 (pseudo t, $^2J_{CP}$ = 15.1, C4), 164.1 (dd, $^1J_{CP}$ = 10.7, $^2J_{CP}$ = 4.6, C5), 191.9 (dd, $^1J_{CP}$ = 56.9, $^2J_{CP}$ = 14.3, C3).

Ethyl 2-(3,4,5-triphenyl-1,2-diphosphacyclopenta-2,4-dienyl)acetate (1b)*.* In a similar manner **1b** was obtained from sodium $(diglyme)_2$ 3,4,5-triphenyl-1,2-diphosphacyclopentadienide (2.62 g, 4.22 mmol) and ethyl-2-chloroacetate $ClCH_2COOEt$ (0.65 g, 5.28 mmol, 25% excess) as a light-yellow oil; yield 1.05 g (60%). ^{1}H NMR (5°C, $CDCl_3$): 0.62 (t, 3H, $^3J_{HH}$ = 7.2, CH_3), 1.33-1.39 (m, 2H, CH_2), 2.04-2.10 (m, 2H, PCH_2), 6.83 (t, 4H, $^3J_{HH}$ = 6.4), 6.96 (d, 4H, $^3J_{HH}$ = 4.8), 7.03 (d, 2H, $^3J_{HH}$ = 5.8), 7.16 (d, 2H, $^3J_{HH}$ = 7.4), 7.23 (d, 2H, $^3J_{HH}$ = 5.4), 7.31 (t, 1H, $^3J_{HH}$ = 8.3). ^{31}P NMR (5°C, $CDCl_3$): 40.5 (d, $^1J_{PP}$ = 389.0), 223.3 (d, $^1J_{PP}$ = 389.0). ^{13}C NMR (5°C, $CDCl_3$): 15.1 (s, CH_3), 31.0 (s, OCH_2), 35.1 (dd, $^1J_{CP}$ = 16.7, $^2J_{CP}$ = 5.8, PCH_2), 126.4 (s, *p*-Ph), 126.5 (s, *p*-Ph), 126.8 (s, *p*-Ph), 127.5 (s, *m*-Ph), 128.0 (s, *m*-Ph), 128.2 (s, *m*-Ph), 128.6 (dd, $^2J_{CP}$ = 11.2, $^3J_{CP}$ = 2.5, *o*-Ph), 129.2 (d, $^3J_{CP}$ = 9.3, *o*-Ph), 131.4 (tr, $^2J_{CP}$ = 9.1, *o*-Ph), 132.1 (s, *o*-Ph), 133.8 (s, $^2J_{CP}$ = 9.4, *o*-Ph), 137.3 (d, $^2J_{CP}$ = 5.4, *ipso*-Ph), 138.3 (dd, $^2J_{CP}$ = 10.4, $^2J_{CP}$ = 4.1, *ipso*-Ph), 142.9 (d, $^2J_{CP}$ = 19.0, *ipso*-Ph), 150.3 (ps.tr., $^2J_{CP}$ = 15.1, C4), 164.1 (dd, $^1J_{CP}$ = 10.8, $^2J_{CP}$ = 4.5, C5), 170.4 (dd, $^2J_{CP}$ = 6.8, $^3J_{CP}$ = 3.5, C=O), 191.90 (dd, $^1J_{CP}$ = 56.8, $^2J_{CP}$ = 14.3, C3).

1-Methoxymethyl-3,4,5-triphenyl-1,2-diphosphacyclopenta-2,4-diene (1c). In a similar manner **1c** was obtained from sodium $(diglyme)_2$ 3,4,5-triphenyl-1,2-

diphosphacyclopentadienide (2.02 g, 3.26 mmol) and chloro(methoxy)methane $ClCH_2OMe$ (0.33 g, 4.08 mmol, 25% excess) as a light-yellow powder; yield 0.69 g (57%); m.p. 59°C. ^{1}H NMR (5°C, $CDCl_3$): 0.89 (s, 3H, CH_3), 1.96-2.01 (m, 2H, CH_2), 6.85 (d, 4H, $^3J_{HH}$ = 3.42), 6.90 (d, 2H, $^3J_{HH}$ = 7.34), 6.96 (d, 4H, $^3J_{HH}$ = 6.85), 6.99-7.03 (m, 1H, $^3J_{HH}$ = 5.38), 7.20 (d, 2H, $^3J_{HH}$ = 7.34), 7.29 (d, 2H, $^3J_{HH}$ = 5.87). ^{31}P NMR (5°C, $CDCl_3$): 61.2 (d, $^1J_{PP}$ = 404.0), 209.3 (d, $^1J_{PP}$ = 404.0). ^{13}C NMR (5°C, $CDCl_3$): 23.4 (d, $^3J_{CP}$ = 7.8, CH_3), 31.5 (dd, $^1J_{CP}$ = 26.3, $^2J_{CP}$ = 15.8, CH_2), 126.4 (s, *p*-Ph), 126.5 (s, *p*-Ph), 126.8 (s, *p*-Ph), 127.5 (s, *m*-Ph), 128.1 (s, *m*-Ph), 128.2 (s, *m*-Ph), 128.6 (s, *o*-Ph), 129.2 (s, *o*-Ph), 130.4 (d, $^3J_{CP}$ = 3.2, *o*-Ph), 137.5 (d, $^3J_{CP}$ = 6.2, *ipso*-Ph), 138.2 (dd, $^3J_{CP}$ = 10.3, $^3J_{CP}$ = 3.7, *ipso*-Ph), 142.8 (d, $^3J_{CP}$ = 19.4, *ipso*-Ph), 149.2 (ps.tr., $^3J_{CP}$ = 14.5, C4), 164.6 (dd, $^1J_{CP}$ = 14.3, $^2J_{CP}$ = 3.1, C5), 187.8 (dd, $^1J_{CP}$ = 57.1, $^2J_{CP}$ = 14.8, C3).

1-(2-Ethoxyethyl)-3,4,5-triphenyl-1,2-diphosphacyclopenta-2,4-diene (1d). In a similar manner **1d** was obtained from sodium $(diglyme)_2$ 3,4,5-triphenyl-1,2-diphosphacyclopentadienide (0.94 g, 1.52 mmol) and 1-bromo-2-ethoxyethane $BrCH_2CH_2OEt$ (0.26 g, 1.90 mmol, 25% excess) as a light-yellow oil; yield 0.36 g (59%). ^{1}H NMR ($CDCl_3$): 0.59 (t, 3H, $^3J_{HH}$ = 6.9, CH_3), 1.42-1.47 (m, 2H, OCH_2), 1.51-1.57 (m, 2H, OCH_2), 1.88-1.93 (m, 2H, PCH_2), 6.85 (d, 4H, $^3J_{HH}$ = 3.42), 6.90 (d, 2H, $^3J_{HH}$ = 7.34), 6.96 (d, 4H, $^3J_{HH}$ = 6.85), 6.99-7.07 (m, 1H, $^3J_{HH}$ = 5.38), 7.20 (d, 2H, $^3J_{HH}$ = 7.34), 7.29 (d, 2H, $^3J_{HH}$ = 5.87). ^{31}P NMR ($CDCl_3$): 51.9 (d, $^1J_{PP}$ = 407.3), 214.1 (d, $^1J_{PP}$ = 407.3). ^{13}C NMR ($CDCl_3$): 16.7 (s, CH_3), 25.6 (s, CH_2), 27.3 (d, $^2J_{CP}$ = 9.7, CH_2), 37.8 (dd, $^1J_{CP}$ = 16.3, $^2J_{CP}$ = 5.8, CH_2), 126.4 (s, *p*-Ph), 126.5 (s, *p*-Ph), 126.8 (s, *p*-Ph), 127.4 (s, *m*-Ph), 128.2 (s, *m*-Ph), 128.3 (s, *m*-Ph), 128.5 (s, *o*-Ph), 129.2 (s, *o*-Ph), 130.5 (d, $^3J_{CP}$ = 1.4, *o*-Ph), 131.7 (s, *o*-Ph), 133.5 (s, *o*-Ph), 137.6 (d, $^3J_{CP}$ = 6.2, *ipso*-Ph), 138.24 (dd, $^3J_{CP}$ = 10.4, $^3J_{CP}$ = 3.8, *ipso*-Ph), 142.8 (d, $^3J_{CP}$ = 9.8, *ipso*-Ph), 149.7

(ps.tr, $^3J_{CP}$ = 15.51, C4), 164.5 (dd, $^1J_{CP}$ = 7.3, $^2J_{CP}$ = 3.1, C5), 185.8 (dd, $^1J_{CP}$ = 47.1, $^2J_{CP}$ = 14.9, C3).

1-Ethyl-3,4,5-tri(*p*-fluorophenyl)-1,2-diphosphacyclopenta-2,4-diene (1e). In a similar manner **1e** was obtained from sodium (diglyme)$_2$ 3,4,5-tri(*para*-flurophenyl)-1,2-diphosphacyclopentadienide (1.86 g, 2.76 mmol) and ethyl iodide EtI (0.54 g, 3.45 mmol, 25% excess) as a light-yellow powder; yield 0.68 g (60%); m.p. 63°C. ^{1}H NMR (CDCl$_3$): 1.14 (dd, $^3J_{HH}$ = 10.3, $^3J_{PH}$ = 8.8, 3H, Me), 1.88-1.93 (m, 2H, CH$_2$), 7.01 (t, 4H, $^3J_{HH}$ = 6.36), 7.13 (d, 4H, $^3J_{HH}$ = 4.89), 7.17 (d, 4H, $^3J_{HH}$ = 7.34). ^{31}P NMR (CDCl$_3$): 73.4 (d, $^1J_{PP}$ = 407.8), 218.5 (d, $^1J_{PP}$ = 407.8). ^{13}C NMR (CDCl$_3$): 12.01 (d, $^2J_{CP}$ = 9.51, CH$_3$), 17.87 (dd, $^1J_{CP}$ = 16.13, $^2J_{CP}$ = 5.79, CH$_2$), 127.4 (s, *p*-C$_{Ph}$), 127.5 (s, *p*-C$_{Ph}$), 127.7 (s, *p*-C$_{Ph}$), 127.9 (s, *m*-C$_{Ph}$), 128.1 (s, *m*-C$_{Ph}$), 128.4 (s, *m*-C$_{Ph}$), 128.7 (dd, $^3J_{CP}$ = 13.4, $^4J_{CP}$ = 2.8, *o*-C$_{Ph}$), 129.4 (d, $^3J_{CP}$ = 9.3, *o*-C$_{Ph}$), 130.5 (t, $^3J_{CP}$ = 7.8, *o*-C$_{Ph}$), 137.8 (d, $^4J_{CP}$ = 5.3, *ipso*-C$_{Ph}$), 139.6 (dd, $^2J_{CP}$ = 11.4, $^3J_{CP}$ = 4.3, *ipso*-C$_{Ph}$), 142.7 (d, $^2J_{CP}$ = 17.2, *ipso*-C$_{Ph}$), 153.2 (t, $^2J_{CP}$ = 14.1, C4), 160.9 (dd, $^1J_{CP}$ = 10.7, $^2J_{CP}$ = 4.8, C5), 161.1 (d, $^1J_{CF}$ = 161.2, C-F), 161.4 (d, $^1J_{CF}$ = 165.0, C-F), 161.4 (d, $^1J_{CF}$ = 163.2, C-F), 176.9 (dd, $^1J_{CP}$ = 24.4, $^2J_{CP}$ = 13.2, C3).

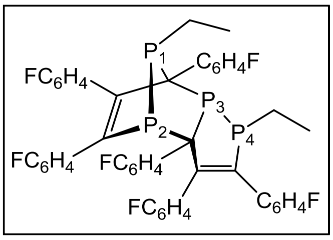

2,3,4,4a,5,6-Hexa(*p*-fluorophenyl)-1-ethyl-1,7,7a-triphospha-4,7-(ethylphosphinidene)-indene (2e). A solution of 0.4 g of 1-ethyl-3,4,5-tri(*para*-fluorophenyl)-1,2-diphosphacyclopenta-2,4-diene (**1e**) in toluene (10 mL) was stirred for 4 h at 60 °C. After cooling to room temp, the solvent was evaporated and the residue was washed with n-hexane, dried in vacuo to leave 0.27 g (67%) to give *rac*-2,3,4,4a,5,6-hexa(*para*-fluorophenyl)-1-ethyl-1,7,7a-triphospha-4,7-(ethylphosphinidene)-indene (**2e**) as a white powder; m.p.130°C. ^{1}H NMR (CDCl$_3$): 0.58

(dt, $^3J_{HH}$ = 7.8, $^3J_{PH}$ = 17.4, 3H, Me), 0.83-0.93 (m, 2H, CH_2), 1.39 (dt, $^3J_{HH}$ = 7.7, $^3J_{PH}$ = 15.9, 3H, Me), 1.92-2.00 (m, 2H, CH_2), 6.34 (t, $^3J_{HH}$ = 8.6, 2H, Ph), 6.67 (t, $^3J_{HH}$ = 8.7, 2H, Ph), 6.69-6.85 (m, 9H, Ph), 6.86-7.23 (m, 7H, Ph), 7.24-7.32 (m, 2H, Ph), 7.41-7.49 (m, 2H, Ph). ^{31}P NMR ($CDCl_3$): -4.4 (d, $^1J_{PP}$ = 226.2, P_1), 1.3 (d, $^1J_{PP}$ = 236.8, P_4), 40.9 (dd, $^1J_{PP}$ = 236.8, $^2J_{PP}$ = 9.6, P_3), 62.5 (dd, $^1J_{PP}$ =226.2, $^2J_{PP}$ = 9.0, P_2). ^{13}C NMR ($CDCl_3$): 10.8 (dd, $^3J_{CP}$ = 2.6, $^2J_{CP}$ = 19.4, Me), 12.4 (dd, $^3J_{CP}$ = 7.3, $^2J_{CP}$ = 13.8, Me), 20.3 (dd, $^2J_{CP}$ = 17.6, $^1J_{CP}$ = 29.6, CH_2), 21.5 (ps.t, $^2J_{CP}$ = 20.1, $^1J_{CP}$ = 21.6, CH_2), 77.0 (dd, $^1J_{CP}$ = 23.6, $^2J_{CP}$ = 7.6, C-PhF), 79.1 (dd, $^1J_{CP}$ = 18.2, $^2J_{CP}$ = 9.5, C-PhF), 114.3 (s, *m*-Ph), 114.5 (s, *m*-Ph), 114.9 (s, *m*-Ph), 115.0 (s, *m*-Ph), 115.1 (s, *m*-Ph), 115.2 (s, *m*-Ph), 115.3 (s, *m*-Ph), 115.4 (s, *m*-Ph), 115.5 (s, *m*-Ph), 115.7 (s, *m*-Ph), 115.9 (s, *m*-Ph), 131.4 (s, *m*-Ph), 132.4 (s, *o*-Ph), 132.5 (s, *o*-Ph), 132.9 (s, *o*-Ph), 133.1 (s, *o*-Ph), 133.3 (s, *o*-Ph), 133.9 (s, *o*-Ph), 134.3 (s, *o*-Ph), 134.5 (s, *o*-Ph), 134.6 (s, o-Ph), 134.9 (s, *o*-Ph), 135.0 (s, *o*-Ph), 135.2 (s, *o*-Ph), 135.4 (s, *o*-Ph), 135.5 (s, *o*-Ph), 136.4 (d, $^3J_{CP}$ = 4.4, *ipso*-Ph), 136.9 (dd, $^2J_{CP}$ = 5.9, $^2J_{CP}$ = 18.4, *ipso*-Ph), 137.5 (d, $^2J_{CP}$ = 9.0, *ipso*-Ph), 137.9 (d, $^2J_{CP}$ = 13.7, *ipso*-Ph), 138.3 (d, $^3J_{CP}$ = 8.3, *ipso*-Ph), 138.7 (dd, $^2J_{CP}$ = 13.5, $^2J_{CP}$ = 8.8, *ipso*-Ph), 139.4 (dd, $^1J_{CP}$ = 14.3, $^2J_{CP}$ = 4.8, C=C), 140.0 (dd, $^1J_{CP}$ = 19.5, $^2J_{CP}$ = 5.4, C=C), 152.9 (d, $^2J_{CP}$ = 14.8, C=C), 155.5 (dd, $^2J_{CP}$ = 15.3, $^2J_{CP}$ = 13.9, C=C), 160.2 (d, $^1J_{CF}$ = 156.4, C-F), 160.7 (d, $^1J_{CF}$ = 151.9, C-F), 161.5 (d, $^1J_{CF}$ = 153.2, C-F), 162.2 (d, $^1J_{CF}$ = 153.4, C-F), 162.8 (d, $^1J_{CF}$ = 152.9, C-F), 163.4 (d, $^1J_{CF}$ = 150.2, C-F). IR (KBr, cm^{-1}): 1017 (s, C-F), 1097 (s, C-F), 1158 (s, C-F). $C_{46}H_{34}F_6P_4$ (825): calcd. C 67.00, H 4.16, P 15.02, F 13.82; found C 66.54, H 4.46, P 15.08, F 13.92.

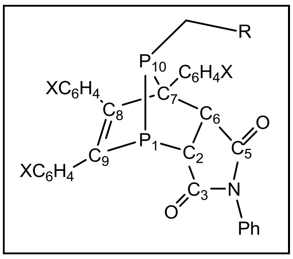

10-Cyanomethylene-4,7,8,9-tetraphenyl-4-aza-1,10-diphosphatricyclo [5.2.1.0^{2,6}] deca-8-ene-3,5-dione (3a). 0.42 g (2.4 mmol, 50% excess) *N*-phenylmaleimide was added to the mixture of **1a** and isomeric cycloadducts **2a** (1:1 mixture, 0.60 g, ~1.63 mmol of 1a) in 10 mL toluene and stirred at 120°C for 25 hours. When the reaction was finished a light-yellow powder had precipitated, which was separated, washed with mixture of 20 ml *n*-hexane and 20 ml toluene, dried in vacuum and recrystallized from hot toluene (5 mL) to give 0.54 g (61%) white powder of 10-cyanomethylene-4,7,8,9-tetraphenyl-4-aza-1,10-diphosphatricyclo [5.2.1.0^{2,6}]deca-8-ene-3,5-dione **3a** with m.p. 128°C. ^{1}H NMR ($CDCl_3$): 1.78 (d, $^2J_{HP}$ = 31.2, 2H, CH_2), 4.24 (dd, $^3J_{HH}$ = 6.38, $^3J_{HP}$ = 10.9, 1H, C6-H), 4.57 (dd, $^2J_{HP}$ = 19.6, $^3J_{HH}$ = 7.2, 1H, C2-H), 6.16 (d, $^3J_{HH}$ = 9.1, 4H, Ph), 7.05 (d, $^3J_{HH}$ = 7.3, 4H, Ph), 7.07 (d, $^3J_{HH}$ = 6.8, 4H, Ph), 7.14 (d, $^3J_{HH}$ = 6.2, 2H, Ph), 7.36 (d, $^3J_{HH}$ = 6.7, 1H, Ph), 7.56-7.61 (m, 5H, Ph). ^{31}P NMR ($CDCl_3$): -28.7 (d, $^1J_{PP}$ = 198.5, P_{10}), 51.4 (d, $^1J_{PP}$ = 198.5, P_1). ^{13}C NMR ($CDCl_3$): 20.1 (dd, $^1J_{CP}$ = 22.2, $^2J_{CP}$ = 14.2, CH_2), 41.6 (s, C6), 47.6 (d, $^1J_{CP}$ = 32.3, C2), 71.7 (dd, $^1J_{CP}$ = 28.3, $^2J_{CP}$ = 4.4, C7), 115.4 (d, $^2J_{CP}$ = 6.4, CN), 125.2 (s, *p*-C_{Ph}), 126.1 (s, *p*-C_{Ph}), 126.8 (s, *p*-C_{Ph}), 127.4 (s, *p*-C_{Ph}), 127.6 (s, *m*-C_{Ph}), 128.1 (s, *m*-C_{Ph}), 128.3 (s, *m*-C_{Ph}), 128.7 (s, *m*-C_{Ph}), 128.9 (s, *o*-C_{Ph}), 129.1 (s, *o*-C_{Ph}), 129.3 (s, *o*-C_{Ph}), 130.1 (s, *o*-C_{Ph}), 130.7 (s, ipso-C_{Ph}), 131.2 (s, *ipso*-C_{Ph}), 135.2 (d, $^2J_{CP}$ = 6.2, *ipso*-C_{Ph}), 136.1 (d, $^1J_{CP}$ = 20.1, *ipso*-C_{Ph}), 139.3 (dd, $^2J_{CP}$ = 7.9, $^2J_{CP}$ = 9.6, C8), 154.4 (dd, $^1J_{CP}$ = 19.1, $^2J_{CP}$ = 4.0, C9), 172.9 (s, C3), 175.6 (s, C5). IR (KBr, cm^{-1}): 1719 (s, CO), 1777 (m, CO), 2211 (s, CN). $C_{33}H_{24}N_2O_2P_2$ (544): calcd. C 73.06, H 4.46, P 11.42, N 5.15; found C 73.35, H 4.76, P 11.16, N 5.00.

10-Ethylacetyl-4,7,8,9-tetraphenyl-4-aza-1,10-diphosphatricyclo[5.2.1.0^{2,6}]deca-8-ene-3,5-dione (3b). In a similar manner **3b** was obtained from mixture of **1b** and

isomeric cycloadducts **2b** (1:1 mixture, 0.55 g, 1.32 mmol of 1b) and excess of *N*-phenylmaleinimide (0.35 g, 2.0 mmol, 50% excess) as a white powder; yield 0.51 g (65%); m.p. 130°C. ^{1}H NMR ($CDCl_3$): 0.72 (t, $^{3}J_{HH}$ = 7.8, 3H, CH_3), 1.92 (q, $^{3}J_{HH}$ = 7.8, 2H, OCH_2), 2.18-2.20 (m, 2H, CH_2), 4.55 (dd, $^{3}J_{HH}$ = 5.4, $^{3}J_{HP}$ = 14.1, 1H, C6-H), 4.77 (dd, $^{3}J_{HH}$ = 6.2, $^{2}J_{HP}$ = 19.6, 1H, C2-H), 6.75 (d, 5H, $^{3}J_{HH}$ = 9.3, Ph), 6.99 (d, 5H, $^{3}J_{HH}$ = 7.3, Ph), 7.07 (d, 5H, $^{3}J_{HH}$ = 6.8, Ph), 7.14 (d, 4H, $^{3}J_{HH}$ = 6.4, Ph), 7.36 (d, 1H, $^{3}J_{HH}$ = 6.8, Ph). ^{31}P NMR ($CDCl_3$): -26.7 (d, $^{1}J_{PP}$ = 198.3, P_{10}), 56.9 (d, $^{1}J_{PP}$ = 198.3, P_1). ^{13}C NMR ($CDCl_3$): 11.2 (s, CH_3), 26.8 (s, CH_2), 30.2 (dd, $^{1}J_{CP}$ = 13.7, $^{2}J_{CP}$ = 6.8, CH_2), 48.7 (s, C2), 51.1 (s, C6), 74.8 (dd, $^{1}J_{CP}$ = 24.3, $^{2}J_{CP}$ = 2.8, C7), 125.8 (s, *p* -C_{Ph}), 126.2 (s, *p*-C_{Ph}), 126.7 (s, *p*-C_{Ph}), 126.8 (s, *p*-C_{Ph}), 127.1 (s, *m*-C_{Ph}), 127.2 (s, *m*-C_{Ph}), 127.4 (s, *m*-C_{Ph}), 127.6 (s, *m*-C_{Ph}), 127.7 (s, *o*-C_{Ph}), 128.4 (s, *o*-C_{Ph}), 129.4 (s, *o*-C_{Ph}), 130.5 (s, *o*-C_{Ph}), 130.8 (s, *ipso*-C_{Ph}), 131.4 (s, *ipso*-C_{Ph}), 138.9 (d, $^{2}J_{CP}$ = 5.3, *ipso*-C_{Ph}), 139.7 (d, $^{1}J_{CP}$ = 20.0, *ipso*-C_{Ph}), 140.9 (dd, $^{1}J_{CP}$ = 27.7, $^{2}J_{CP}$ = 19.2, C8), 157.9 (dd, $^{2}J_{CP}$ = 18.3, $^{2}J_{CP}$ = 4.1, C9), 174.9 (s, C3), 177.9 (s, C5), 185 (d, $^{2}J_{CP}$ = 9.8, C(O)O). IR (KBr, cm^{-1}): 1713 (s, CO), 1773 (m, CO), 1800 (s, C(O)O). $C_{35}H_{29}NO_4P_2$ (590): calcd. C 71.30, H 4.96, P 10.51, N 2.38; found C 71.35, H 4.76, P 10.76, N 2.37.

10-Methoxymethyl-4,7,8,9-tetraphenyl-4-aza-1,10diphosphatricyclo[5.2.1.0^{2,6}]deca-8-ene-3,5-dione (3c). In a similar manner **3c** was obtained from mixture of **1c** and isomeric cycloadducts **2c** (1:1 mixture, 0.41 g, 1.09 mmol of 1c) and excess of *N*-phenylmaleinimide (0.27 g, 1.64 mmol, 50 % excess) as a white powder; yield 0.37 g (62%); m.p. 134°C. ^{1}H NMR ($CDCl_3$): 0.86 (s, 3H, CH_3), 2.34-2.37 (m, 2H, CH_2), 4.23 (dd, $^{3}J_{HH}$ = 6.1, $^{3}J_{HP}$ = 12.2, 1H, C6-H), 4.34 (dd, $^{3}J_{HH}$ = 5.2, $^{2}J_{HP}$ = 17.3, 1H, C2-H), 6.75 (d, 5H, $^{3}J_{HH}$ = 9.3, Ph), 6.99 (d, 5H, $^{3}J_{HH}$ = 7.3, Ph), 7.07 (d, 4H, $^{3}J_{HH}$ = 6.9, Ph), 7.14 (d, 4H, $^{3}J_{HH}$ = 6.4, Ph), 7.36 (d, 2H, $^{3}J_{HH}$ = 6.9, Ph). ^{31}P NMR ($CDCl_3$): -25.1 (d, $^{1}J_{PP}$ = 200.3, P_{10}), 68.3 (d, $^{1}J_{PP}$ = 200.3, P_1). ^{13}C NMR ($CDCl_3$): 15.1 (d, $^{3}J_{CP}$ = 3.9,

CH_3), 30.3 (dd, ${}^1J_{CP}$ = 23.8, ${}^2J_{CP}$ = 5.9, CH_2), 43.3 (dd, ${}^1J_{CP}$ = 33.0, ${}^2J_{CP}$ = 14.6, C2), 44.5 (d, ${}^2J_{CP}$ = 16.9, C6), 71.4 (dd, ${}^1J_{CP}$ = 22.5, ${}^2J_{CP}$ = 6.7, C7), 126.0 (s, *p*-C_{Ph}), 127.3 (s, *p*-C_{Ph}), 127.6 (s, *p*-C_{Ph}), 127.8 (s, *p*-C_{Ph}), 127.9 (s, *m*-C_{Ph}), 128.4 (s, *m*-C_{Ph}), 128.9 (s, *m*-C_{Ph}), 129.4 (s, *m*-C_{Ph}), 129.5 (s, *o*-C_{Ph}), 129.7 (s, *o*-C_{Ph}), 129.9 (s, *o*-C_{Ph}), 130.0 (s, *o*-C_{Ph}), 130.7 (s, *ipso*-C_{Ph}), 131.4 (s, *ipso*-C_{Ph}), 134.2 (s, *ipso*-C_{Ph}), 136.1 (d, ${}^1J_{CP}$ = 5.1, *ipso*-C_{Ph}), 139.1 (dd, ${}^2J_{CP}$ = 23.1, ${}^2J_{CP}$ = 2.6, C8), 141.3 (dd, ${}^1J_{CP}$ = 23.1, ${}^2J_{CP}$ = 2.6, C9), 168.7 (d, ${}^2J_{CP}$ = 10.5, C3), 174.6 (d, ${}^3J_{CP}$ = 14.8, C5). IR (KBr, cm^{-1}): 1714 (s, CO), 1774 (m, CO). $C_{33}H_{27}NO_3P_2$ (548): calcd. C 72.39, H 4.97, P 11.31, N 2.56; found C 72.35, H 4.86, P 11.46, N 2.47.

10-Ethyl-7,8,9-tri(*para*-fluorophenyl)-4-aza-4-phenyl-1,10-diphosphatricyclo-[5.2.1.0^{2,6}] deca-8-ene-3,5-dione (3e). In a similar manner **3e** was obtained from cycloadduct *rac*-2,3,4,4a,5,6-hexa(*para*-fluorophenyl)-1-ethyl-1,7,7a-triphospha-4,7-(ethylphosphinidene)-indene (**2e**) (0.16 g, 0.19 mmol) and excess of *N*-phenylmaleinimide (0.06 g, 0.29 mmol, 50 % excess) as a white powder; yield 0.12 g (55%); m.p. 130°C. ^{1}H NMR ($CDCl_3$): 0.72 (t, 3H, ${}^3J_{HH}$ = 7.8, CH_3), 1.32-1.35 (m, 2H, CH_2), 4.43 (dd, ${}^3J_{HH}$ = 5.4, ${}^2J_{HP}$ = 14.1, 1H, C6-H), 4.77 (dd, ${}^3J_{HH}$ = 6.3, ${}^2J_{HP}$ = 15.2, 1H, C2-H), 6.75 (d, 4H, ${}^3J_{HH}$ = 9.3, Ph), 6.99 (d, 4H, ${}^3J_{HH}$ = 7.3, Ph), 7.07 (d, 4H, ${}^3J_{HH}$ = 6.8, Ph), 7.14 (d, 3H, ${}^3J_{HH}$ = 6.4, Ph), 7.36 (d, 2H, ${}^3J_{HH}$ = 6.8, Ph). ^{31}P NMR ($CDCl_3$): -28.1 (d, ${}^1J_{PP}$ = 196.5, P_{10}), 80.3 (d, ${}^1J_{PP}$ = 196.5, P_1). ^{13}C NMR ($CDCl_3$): 11.1 (d, ${}^2J_{CP}$ = 7.8, CH_3), 30.5 (dd, ${}^1J_{CP}$ = 23.7, ${}^2J_{CP}$ = 8.8, CH_2), 48.7 (s, C2), 51.1 (s, C6), 74.8 (dd, ${}^1J_{CP}$ = 24.3, ${}^2J_{CP}$ = 2.8, C7), 125.9 (s, *p*-C_{Ph}), 126.4 (s, *p*-C_{Ph}), 126.6 (s, *p*-C_{Ph}), 126.7 (s, *p*-C_{Ph}), 127.1 (s, *m*-C_{Ph}), 127.2 (s, *m*-C_{Ph}), 127.4 (s, *m*-C_{Ph}), 127.5 (s, *m*-C_{Ph}), 127.6 (s, *o*-C_{Ph}), 128.3 (s, *o*-C_{Ph}), 129.3 (s, *o*-C_{Ph}), 130.4 (s, *o*-C_{Ph}), 130.6 (s, *ipso*-C_{Ph}), 131.7 (s, *ipso*-C_{Ph}), 138.8 (d, ${}^2J_{CP}$ = 5.2, *ipso*-C_{Ph}), 139.2 (d, ${}^1J_{CP}$ = 20.7, *ipso*-C_{Ph}), 138.5 (dd, ${}^1J_{CP}$ = 21.7, ${}^2J_{CP}$ = 18.3, C8), 154.9 (dd, ${}^2J_{CP}$ = 18.3, ${}^2J_{CP}$ = 4.1, C9), 162.2 (d, ${}^1J_{CF}$ =

154.4, C-F), 162.4 (d, $^{1}J_{CF}$ = 156.9, C-F), 162.5 (d, $^{1}J_{CF}$ = 160.2, C-F), 174.9 (s, C3), 177.99 (s, C5). IR (KBr, cm^{-1}): 1023 (s, C-F), 1063 (s, C-F), 1118 (s, C-F), 1714 (s, CO), 1773 (m, CO). $C_{33}H_{24}F_3NO_2P_2$ (585): calcd. C 67.70, H 4.13, P 10.58, F 9.73, N 2.39; found C 67.50, H 4.33, P 10.38, F 9.83, N 2.49.

References

1. Bruker APEX2 Software Suite for Crystallographic Programs, Bruker AXS, Inc., Madison, WI, USA, **2009**.

2. Bruker Area detector control and integration software. Version 5.x. In: SMART and SAINT. Madison, Wisconsin (USA): Bruker Analytical X-ray Instruments Inc.; **1996**.

3. Sheldrick, G. M. SADABS: Programs for Crystal Structure Analysis, University of Göttingen, Institut für Anorganische Chemie der Universität, Tammanstrasse 4, D-3400 Göttingen, Germany, **1997**.

4. Sheldrick, G. M. *Acta Crystallogr*. **2008**, 64, 112-122.

5. Farrugia, L. J. *J. Appl. Cryst*. **1997**, 30, 565-566.

6. Macrae, F.; Bruno, I. J.; Chisholm, J.A.; Edgington, P.R.; McCabe, P.; Pidcock, E.; Rodriguez-Monge, L.; Taylor, R.; Streek J. van de; Wood, P. A. *J. Appl. Cryst.* **2008**, 41, 466-470.

7. Spek, A. L. *Acta Crystallogr., Sect. D: Biol. Crystallogr*. **2009**, 65, 148-155.

8. Bezkishko I.; Miluykov V.; Kataev A.; Krivolapov D.; Litvinov I.; Sinyashin O.; Hey-Hawkins E. *J. Organomet. Chem*. **2008**, 693, 3318–3320.

9. Bezkishko I.; Miluykov V.; Sinyashin O.; Hey-Hawkins E.; *Phosphorus, Sulfur, and Silicon and the Rel. Elem*., **2011**, 186, 657–659.
